# Remarks on the Medical Treatment of Cataract

**Published:** 1848-07

**Authors:** James Bryan

**Affiliations:** Lecturer on Surgery, Honorary Member of the Philadelphia Medical Society, &c.


### MEDICAL EXAMINER,

AND

### RECORD OF MEDICAL SCIENCE.

NEW SERIES.—No. XLIII.—JULY, 1848.

### ORIGINAL COMMUNICATIONS.

*Remarks on the Medical Treatment of Cataract.* By JAMES BRYAN, M. D. Lecturer on Surgery, Honorary Member of the Philadelphia Medical Society, &c.

It is generally conceded that every successful application of therapeutic measures, which results in diminishing the number of diseases which demand the intervention of the knife, is an advance in the healing art; and that the necessity for the use of the knife is evidence of the imperfection of medical science. I propose, in the following remarks, to present some cases and opinions which tend to prove that cataract, in some forms at least, and particularly in its early stages, is curable without the aid of an operation.

I am well aware of the fact, that the editor of a respectable journal, in conversing upon this matter with me, when he said that a case reported by one of my friends was "all humbug," did but express the sentiments of a large body, if not the whole of the medical profession on this subject. Nevertheless, that our forefathers have generally neglected or condemned this practice is no reason why we should do so likewise. Sad is the prospect of advancement in our noble art, if every attempt at improvement is to be frowned down by '*humbug*.' This is emphatically a day of free thought, and free discussion, and every man has a right to be heard. But to our subject.

In studying some cases of incipient and more advanced cataract, which presented themselves at my surgical clinique in the Philadelphia Dispensary, in the years 1841–2–3 and 4, I was led, through the following course of reasoning, to adopt the medical plan of treatment in those cases in preference to the surgical. Three of the cases were, respectively, a cook, a washerwoman, and a chamber-maid. The cook was exposed to the influence of a large fire in one of our hotels, and had been so for a number of years. This position is similar, of course, to that of men who work at forges, iron works, at the anvil, &c. &c., of which England presents so many who suffer from cataract, and of which this country is not deficient in numbers. The washerwoman was exposed to heated vapour, and the fire accompanying her occupation. And the chamber-maid had been exposed to neither of these influences.

While a student, I had been in the habit of dissecting the eyes of different animals, such as those of the ox, the pig, the sheep, and the calf, for anatomical and physiological purposes; and I observed that the eyes of the calf were invariably found to contain the lens in a greater or lesser degree of opacity; the others were not. The calf, I knew, previous to death, was suspended by the hind legs, for the purpose of more effectually draining the sanguineous, particularly the venous system. The eye, in this position, must, in the young animal, necessarily be engorged, and this I conceived to be the cause of the cataract. Inflammatory action, induced by the causes acting in the above cases, and all similar ones, must most probably be a cause of cataract, especially if we consider a state of engorgement of the capsule of the lens as producing the same disease. With this view of the nature and cause of cataract I was prepared to stop the growing, and perhaps remove the already formed opacity of the lens, by antiphlogistics, counter-irritation, and alteratives. The first patient, Anna Jones, aged 48 years, with cataract in both eyes, was placed under this treatment. Having a strong and full pulse, she was first bled pretty freely; then leeches, six in number, were directed to be applied to the external angles of the eyes and to the lower lids.

A free catharsis was induced by senna and sulphate of magnesia; and the action on the bowels kept up until the next clinique day, by taking each morning a wine-glassful of the infusion. Blisters were then directed to the temples, and an ointment, consisting of ungt. resinæ ℥ii, with cerat. cantharid. ʒii, used to dress the blistered surface. The eyes to be covered with green silk shades, and the patient to remain in a dark room. A little of the extract of belladonna was applied to the eye-lids every evening, The heat of fire, the dust, and high winds to be avoided. At the end of the first week we began the use of the blue mass in three grain pills three times a day, and continued it until the gums were 'touched.' A mild collyrium of acetate of lead, three grains to the ounce of water, was applied to the eyes. Eight days after we began the use of the blue mass, the gums were sore, and the sight began sensibly to improve. The blisters were kept sore for two weeks longer, when they were allowed to heal, the sight having become entirely restored, and the opacity having disappeared in toto.

The second case was that of Mrs. C., aged 40, a washerwoman, of a good constitution. In her the disease had come on gradually, and was not perceived until one eye had become nearly blind, and the other began to be affected. The second eye was but slightly opaque when she came to the clinique. That of the other eye was equally distinct with those of the last case.

The treatment in this case was a repetition of that adopted in the former, with the advantage of terminating in complete restoration of vision in a shorter time.

The third case was Miss M. B., a young, healthy chambermaid, eighteen years of age, whose cataract had been in progress without any known cause, for about three months, and was confined entirely to one eye. In this case the venesection was omitted.

The rest of the treatment was resorted to with the same success in about four weeks.

In my lectures on diseases of the eyes, I have been in the habit of mentioning these and other cases to my class, with the view of obtaining further information on the subject, and inducing them to try the treatment.

One of my last year's class, Dr. W. G. Smith, of New Hampshire, whose industry, intelligence and integrity are known best by those who know him most intimately, and who has practised medicine some seven or eight years, writes me as follows in reference to this mode of treatment, which he has tried in the case of his aged mother.

GREAT FALLS, April 17, 1848.

*Dear Doctor,*—I write to inform you that I arrived home safely one week after I left Philadelphia. I found my wife and child well, but my mother I found had lost the sight of her left eye by cataract, for about two months, and could see but very little with the right one. On examining them I felt convinced that the lens of the right eye was opaque, and of a pearl colour. I then remembered what you said in your lectures on the eye, about those cases which you cured without an operation. As well as I could recollect, your patients were in a similar condition to my mother. I immediately put on four leeches on each temple, and blisters behind the ears. I applied the leeches every second or third day for two weeks, and kept the blisters discharging the whole time. I then ceased leeching, and allowed the blisters behind the ears to heal, and placed one large one on the back of the neck, which I kept discharging for several days. I also gave her a laxative and an alterative—extract of butternut, hydrarg. cum creta, every night, combined with two grains of quinine—which I continued until the mouth became a little sore. I then left off the first two articles, and gave her one grain of sulphate of quinia three times a day for one week afterwards. *She can now see perfectly well* with her right eye, and all pain has left the other one, but she cannot see with it, except to distinguish day from night; sometimes she says she can see her hand with it.

N. B.—I treated both eyes alike. I should have written to you before, but I have been waiting to see how the above case would terminate. My being able to save my mother's sight in her old age, compensates me for all the time and expense of last winter.

Your obedient servant,

W. G. SMITH.

To Dr. JAMES BRYAN, N. E. cor. 10th and Arch streets, Philada.

The ratio of cures in these few cases seems to me to warrant a further trial of the practice. I have not the statistics at hand of the cures of cataract by the usual surgical operations, but this is well known, that we seldom operate until both eyes are involved, and consider ourselves as having done tolerably well if we secure the healthy use of *one* by an operation on both; at least the result of the practice, as far as I have observed it, goes to show that after waiting until the cataract *ripens*, and losing all that time, a very large percentage of the cases operated upon are content to gain vision in one eye.

